# Separation of type and grade in cervical tumours using non-mono-exponential models of diffusion-weighted MRI

**DOI:** 10.1007/s00330-016-4417-0

**Published:** 2016-05-24

**Authors:** Jessica M. Winfield, Matthew R. Orton, David J. Collins, Thomas E. J. Ind, Ayoma Attygalle, Steve Hazell, Veronica A. Morgan, Nandita M. deSouza

**Affiliations:** 1 MRI Unit, The Royal Marsden NHS Foundation Trust, Downs Road, Sutton, Surrey SM2 5PT UK; 2 Cancer Research UK Cancer Imaging Centre, Division of Radiotherapy and Imaging, The Institute of Cancer Research, 123 Old Brompton Road, London, SW7 3RP UK; 3 Gynaecology Unit, The Royal Marsden NHS Foundation Trust, Fulham Road, London, SW3 6JJ UK; 4 Department of Histopathology, The Royal Marsden NHS Foundation Trust, Fulham Road, London, SW3 6JJ UK

**Keywords:** Diffusion-weighted magnetic resonance imaging, Analysis, regression, Apparent diffusion coefficient, Intravoxel incoherent motion (IVIM), Cervical cancer

## Abstract

**Objectives:**

Assessment of empirical diffusion-weighted MRI (DW-MRI) models in cervical tumours to investigate whether fitted parameters distinguish between types and grades of tumours.

**Methods:**

Forty-two patients (24 squamous cell carcinomas, 14 well/moderately differentiated, 10 poorly differentiated; 15 adenocarcinomas, 13 well/moderately differentiated, two poorly differentiated; three rare types) were imaged at 3 T using nine b-values (0 to 800 s mm^-2^). Mono-exponential, stretched exponential, kurtosis, statistical, and bi-exponential models were fitted. Model preference was assessed using Bayesian Information Criterion analysis. Differences in fitted parameters between tumour types/grades and correlation between fitted parameters were assessed using two-way analysis of variance and Pearson's linear correlation coefficient, respectively.

**Results:**

Non-mono-exponential models were preferred by 83 % of tumours with bi-exponential and stretched exponential models preferred by the largest numbers of tumours. Apparent diffusion coefficient (ADC) and diffusion coefficients from non-mono-exponential models were significantly lower in poorly differentiated tumours than well/moderately differentiated tumours. α (stretched exponential), K (kurtosis), *f* and D* (bi-exponential) were significantly different between tumour types. Strong correlation was observed between ADC and diffusion coefficients from other models.

**Conclusions:**

Non-mono-exponential models were preferred to the mono-exponential model in DW-MRI data from cervical tumours. Parameters of non-mono-exponential models showed significant differences between types and grades of tumours.

***Key Points*:**

• *Non-mono-exponential DW-MRI models are preferred in the majority of cervical tumours*.

• *Poorly differentiated cervical tumours exhibit lower diffusion coefficients than well/moderately differentiated tumours*.

• Non-mono-exponential model parameters α, K, f, and D* differ between tumour types.

• *Micro-structural features are likely to affect parameters in non-mono-exponential models differently*.

## Introduction

It is increasingly recognised that the observed diffusion-weighted magnetic resonance imaging (DW-MRI) signal attenuation in biological tissues is not completely described by a Gaussian process [1–5]. The use of non-mono-exponential models provides a better description of the DW-MRI signal, and parameters derived from these models allow more detailed investigation of differences between tumour sub-types or inter-tumour heterogeneity [6–11] and may also provide an earlier indication of response to treatment [12, 13]. However, use of a model with a large number of additional parameters risks over-fitting the data and may be sensitive to noise characteristics of the system rather than structural properties of the tumour or normal tissue. The potential for exploiting these parameters to describe tumour phenotypes remains substantial, but data relating them to the micro-structural properties of tumours or normal tissues is limited, particularly in body applications. Owing to the complexity of tissue and tumour micro-structure within a voxel [14], these models can at least be viewed as phenomenological descriptions of the data and in addition have demonstrable value as empirical markers of tissue status [15]. Nevertheless, their relationship to tissue micro-structural properties is an important topic of ongoing exploration [16, 17].

In cervical cancer the lower apparent diffusion coefficient (ADC) in tumours compared with non-tumour epithelium provides excellent tumour-to-normal-tissue contrast and DW-MRI is routinely used in conjunction with T_2_-weighted imaging for tumour detection [18–20]. Previous studies have shown lower ADCs in poorly differentiated tumours than in well/moderately differentiated tumours, but attempts to distinguish between squamous cell carcinoma and adenocarcinoma using ADC estimates have yielded mixed results [21, 22]. Use of a bi-exponential model has indicated a lower perfusion fraction (*f*) and diffusion coefficient (D) in cervical tumours than in normal cervix [23]. Assessment of the relationships between fitted parameters of DW-MRI models and tumour histopathology would establish the ability of a model to detect differences between grades and types of tumour, and the potential to detect treatment effects that may not be described by the ADC derived from the mono-exponential model. The aims of this exploratory study, therefore, were to assess the performance of mono-exponential and non-mono-exponential models of the DW-MRI signal in cervical tumours and assess whether fitted parameters from these models can be used to distinguish between types and grades of tumours.

## Materials and methods

### Patients

Forty-two consecutive patients with histologically proven cervical tumours and tumour volume at least 50 mm^3^ visible on DW-MRI, recruited over two years (from May 2013 to May 2015), were included in this prospective single-centre study. The study was approved by a national research ethics committee. All patients gave their written consent to participate in this study. Tumours included 24 squamous cell carcinomas (14 well or moderately differentiated, 10 poorly differentiated), 15 adenocarcinomas (13 well or moderately differentiated, two poorly differentiated), one clear cell carcinoma, one poorly differentiated carcinoma with focal squamous differentiation and for the large part neuroendocrine differentiation, and one high grade carcinoma that could not be sub-classified. The latter three tumours were included in the assessment of models, but excluded from the analysis of grades and types.

### Imaging protocol

Each patient underwent one MRI examination. Hyoscine butylbromide (20 mg) i.m. was administered before scanning to reduce image artefacts due to peristalsis. Patients were scanned on a Philips Achieva 3 T MR scanner using an endovaginal coil, as described previously, engineered for imaging at 3 T [24]. Following T_2_-weighted and DW images acquired transversely, coronally, and sagitally through the cervix, a sequence with nine b-values between 0 s mm^-2^ and 800 s mm^-2^ was acquired coronally through the cervix for assessment of DW-MRI models. The protocol for this sequence was as follows: single-shot EPI; FOV = 100 mm x 100 mm; PE direction = RL; acquired matrix (read) = 80; reconstructed matrix (read) = 224; acquired pixel size = 1.25 mm x 1.25 mm; slice thickness = 2 mm; slice gap = 0.1 mm; 24 slices; TE = 52 ms; TR = 6500 ms; b = 0, 20, 40, 60, 80, 100, 300, 500, 800 s mm^-2^; Δ/δ = 25.5/7.5 ms; SPIR fat suppression; NSA = 1 for b-values < 500 s mm^-2^; NSA = 2 for b-values ≥ 500 s mm^-2^; total acquisition time = 7 min 9 s.

### Analysis

Regions of interest (ROIs) were drawn on computed b = 800 s mm^-2^ DW images [25] using in-house software, with reference to the T_2_-weighted images. ROIs were drawn on all slices on which the tumour appeared. The median tumour volume was 2.3 cm^3^ (range 0.07 cm^3^ to 40.8 cm^3^). Mono-exponential (Eq. 1), stretched exponential (Eq. 2), kurtosis (Eq. 3), statistical (Eq. 4), and bi-exponential (Eq. 5) models were fitted to the data at each pixel using all nine b-values using least-squares fits (Matlab 2014a, MathWorks Inc., Natick, MA, USA).

These five models, recently applied in DW-MRI in body applications, were investigated. The mono-exponential model (Eq. 1), is commonly applied in DW-MRI to estimate the ADC.

1 $$ S={S}_0 \exp \left(-b\mathrm{A}\mathrm{D}\mathrm{C}\right) $$

The stretched exponential model (Eq. 2) uses a distributed diffusion coefficient (DDC) and “stretching parameter” (α) to describe non-mono-exponential decay curves [2]. When fitting the stretched exponential model, the “stretching parameter”, α, was constrained to lie between 0 and 1.

2 $$ S={S}_0 \exp \left(-{\left(b\mathrm{D}\mathrm{D}\mathrm{C}\right)}^{\alpha}\right) $$

The kurtosis model (Eq. 3) uses the first two terms of a cumulate expansion to describe non-mono-exponential decay of a DW-MRI signal [4].

3 $$ S={S}_0 \exp \left(-b{\mathrm{D}}_{\mathrm{K}}+\frac{1}{6}{b}^2{\mathrm{D}}_{\mathrm{K}}^2\mathrm{K}\right) $$

The statistical model (Eq. 4) describes the distribution of diffusion coefficients within a voxel using a truncated Gaussian distribution with mode D_s_ and scale parameter σ, where Φ is the error function [3]. In this study, the fitted parameters D_s_ and σ were also reformulated to obtain the mean (D_s'_) and standard deviation (σ') of the distribution of diffusion coefficients within a voxel using results derived elsewhere for a truncated normal distribution [26].

4 $$ S={S}_0\left(\frac{1+\varPhi \left(\frac{{\mathrm{D}}_{\mathrm{s}}}{\sigma \sqrt{2}}-\frac{b\sigma }{\sqrt{2}}\right)}{1+\varPhi \left(\frac{{\mathrm{D}}_{\mathrm{s}}}{\sigma \sqrt{2}}\right)}\right) \exp \left(-b{\mathrm{D}}_{\mathrm{s}}+\frac{1}{2}{b}^2{\sigma}^2\right) $$

The bi-exponential model (Eq. 5), also called intravoxel incoherent motion (IVIM), describes a fast component of the DW-MRI signal (D*), associated with perfusion, and a slow component (D), associated with diffusion [1]. The term *f* in Eq. 5 describes the perfusion fraction. When fitting the bi-exponential model, starting values of D, *f,* and D* were determined from a least-squares fit of a mono-exponential curve to the signal at the highest three b-values and another mono-exponential curve fitted to the remaining signal at the lower b-values; these starting values were used for the least-squares fit of the bi-exponential curve to the data at all nine b-values.

5 $$ S={S}_0\left(f \exp \left(-b{\mathrm{D}}^{*}\right)+\left(1-f\right) \exp \left(-b\mathrm{D}\right)\right) $$

The diffusion coefficients from the non-mono-exponential models (DDC, D_K_, D_s_, D_s'_, and D) may provide comparable information to ADC. For all the above models the signal is expected to decrease with increasing b-value; therefore, any pixels where the signal increased between any pair of successive b-values were excluded from the analysis. Pixels with a fitted S_0_ below a threshold value in mono-exponential fits were also excluded; a threshold of 20 was chosen as this was the mean pixel value in background regions at the edges of the images far from the coil. The median number of pixels included in the analysis per tumour was 4411 (range 56 to 56,722). The median value of each fitted parameter, calculated for each tumour, was used for subsequent analysis in order to reduce sensitivity to outlier values. Statistical preference for the five models was quantified using the Bayesian information criterion (BIC), which penalises additional parameters in the models, as described in Eq. 6 where *L(θ)* is the value of the maximised likelihood objective function for a model with *k* parameters fit to *N* data points [27].

6 $$ \mathrm{B}\mathrm{I}\mathrm{C}=-2 \log L\left(\theta \right)+k \log (N) $$

The preferred model, defined as the model with the lowest (i.e. most negative) BIC, was determined for each pixel. The preferred model for each tumour was defined as the model preferred by the largest number of pixels in the tumour. Two-way analysis of variance (ANOVA, Matlab 2014a) was used to assess differences between types and grades of tumours for each of the fitted parameters. No correction for multiple comparisons was applied owing to the expected correlation between many of the fitted parameters. Pearson's linear correlation coefficient (Matlab 2014a) was used to assess correlation between ADC and diffusion coefficients from non-mono-exponential models and to assess correlation between parameters within each non-mono-exponential model.

Tumour grade and type were determined from histopathological analysis of post-surgery samples (n = 12) or pre-imaging cone biopsy data (n = 30), which samples a substantial (∼1 cm^3^) tumour volume.

## Results

**Fig. 1:**
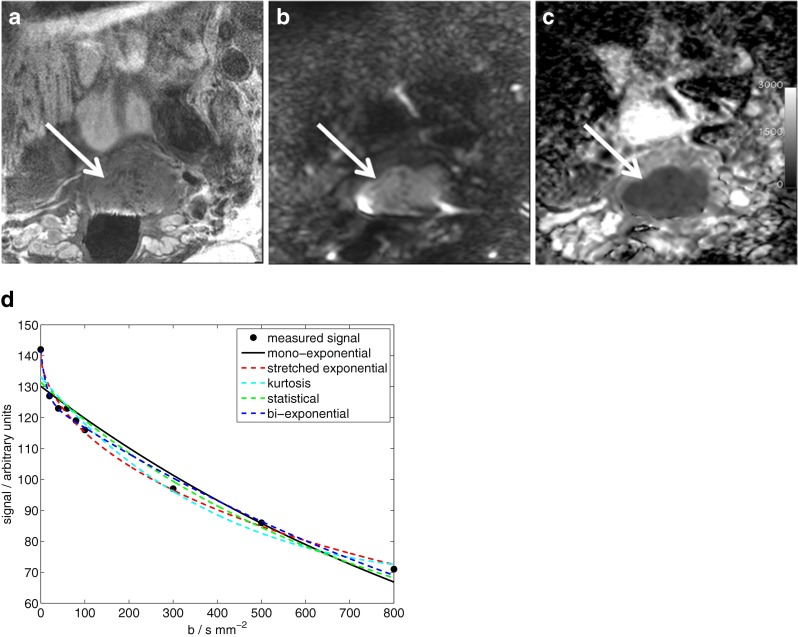
**a** T_2_-weighted image, **b** diffusion-weighted image (b = 800 s mm^-2^), **c** ADC map from a well-differentiated squamous cell carcinoma. **d** Measured signal and fitted curves from one pixel near the centre of the tumour shown in (**a**-**c**)

The well-differentiated squamous cell carcinoma visualised in Fig. 1 demonstrates restricted diffusion (bright signal on the diffusion-weighted image and corresponding dark region on the ADC). Example curve fits from one pixel (Fig. 1d) show clear non-mono-exponential signal attenuation with increasing b-value, which is better described by the non-mono-exponential models (dashed lines in Fig. 1d) than the mono-exponential model (solid black line in Fig. 1d).

The percentages of tumours where BIC analysis indicated that the bi-exponential and stretched exponential models were preferred by the majority of pixels were 43 % and 36 %, respectively (Table 1). Overall, non-mono-exponential models were preferred by 83 % of tumours, with the mono-exponential model preferred by only 17 %. When looking at the number of tumours of each type and grade where each model was preferred, there is no clear preference of a particular type or grade for any of the models. When considering the tumours where each of the models was preferred, the percentage of pixels preferring that model ranged from 26 % to 48 %. This indicates a moderate preference for the dominant model, but also demonstrates that there are many pixels within each tumour that prefer models other than the dominant model.

**Table 1:** Percentage of tumours where mono-exponential, stretched exponential, kurtosis, statistical or bi-exponential models were preferred by the largest number of pixels

Model	Percentage (number) of tumours where model preferred	Numbers of tumours of each type and grade where model preferred	Median (range) percentage of pixels where model was preferred, considering only tumours where model was preferred overall (%)
Mono-exponential	17 (7)	1 squamous cell carcinoma (well/moderately differentiated), 6 adenocarcinomas (all well/moderately differentiated)	32 (26 to 36)
Stretched exponential	36 (15)	7 squamous cell carcinomas (3 well/moderately differentiated, 4 poorly differentiated), 6 adenocarcinomas (5 well/moderately differentiated, 1 poorly differentiated), 2 others	36 (28 to 48)
Kurtosis	5 (2)	2 adenocarcinomas (1 well/moderately differentiated, 1 poorly differentiated)	36 (36 to 37)
Statistical	0 (0)	n/a	n/a
Bi-exponential	43 (18)	16 squamous cell carcinomas (10 well/moderately differentiated, 6 poorly differentiated), 1 adenocarcinoma (well/moderately differentiated), 1 other	37 (31 to 48)

Figures in brackets show the numbers of tumours where each model was preferred. The numbers of tumours of each type and grade where each model was preferred are also noted

**Fig. 2:**
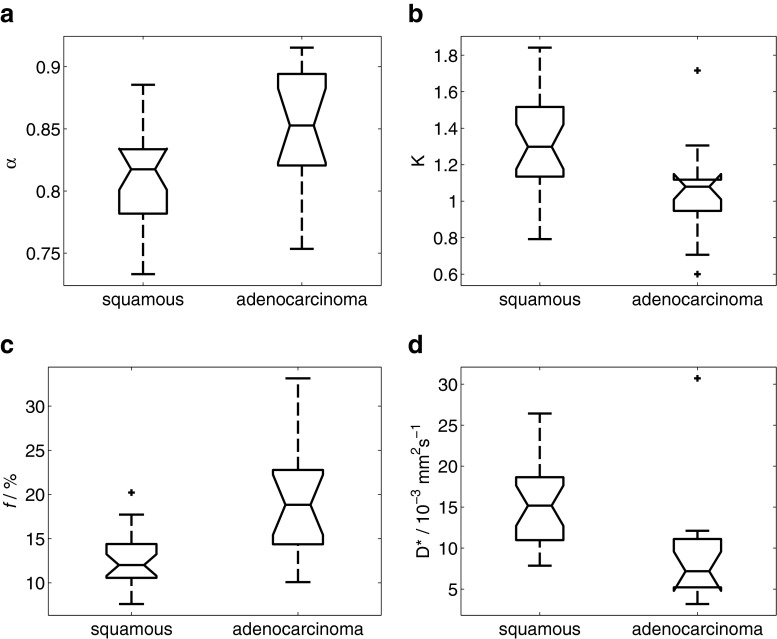
Differences between types of tumour (squamous cell carcinoma (n = 24) versus adenocarcinoma (n = 15)) in (**a**) α from the stretched exponential model, **b** K from the kurtosis model, and **c** *f* and **d** D* from the bi-exponential model

**Fig. 3:**
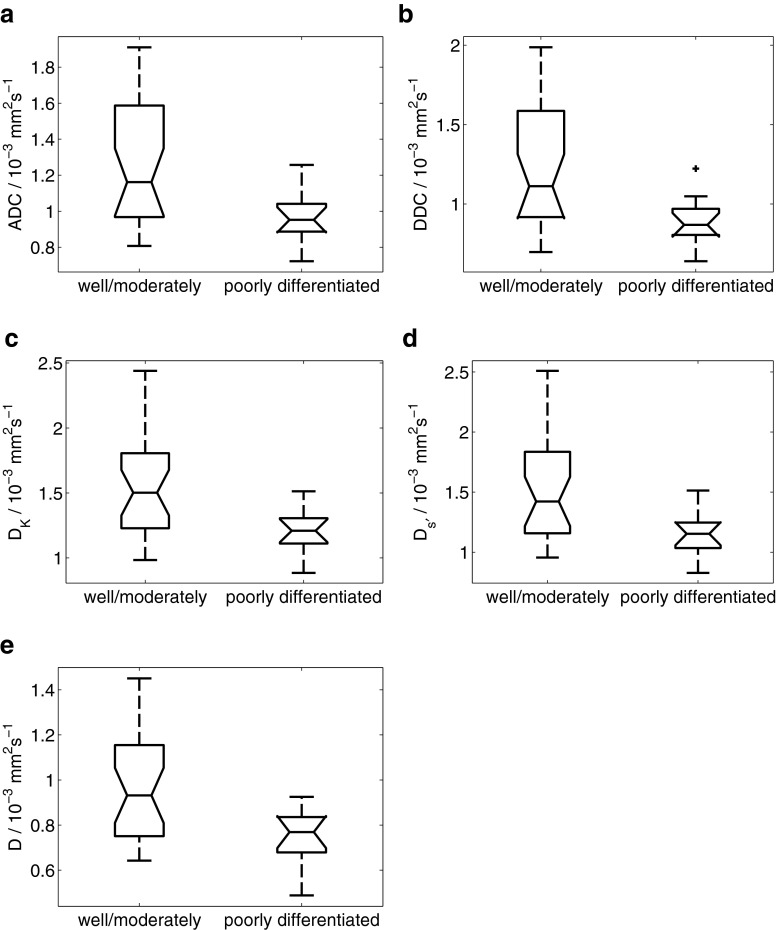
Differences between grades of tumour (well/moderately differentiated (n = 27) versus poorly differentiated (n = 12)) in **a** ADC from the mono-exponential model, **b** DDC from the stretched exponential model, **c** D_K_ from the kurtosis model, **d** D_s'_ from the statistical model, and **e** D from the bi-exponential model

Results from ANOVA, summarised in Table 2, showed that α from the stretched exponential model, K from the kurtosis model, and *f* and D* from the bi-exponential model were significantly different between types of tumour (squamous cell carcinoma versus adenocarcinoma) (Fig. 2). ADC from the mono-exponential model, DDC from the stretched exponential model, D_K_ from the kurtosis model, D_s'_ from the statistical model and D from the bi-exponential model were significantly different between tumour grades (well/moderately differentiated versus poorly differentiated) (Fig. 3).

**Table 2:** Assessment of differences in each fitted parameter between types and grades of tumour using two-way ANOVA. (s.d. standard deviation) * *p* < 0.05

Model	Parameter	*p*-value from ANOVA (type)	*p*-value from ANOVA (grade)
Mono-exponential	ADC	0.1	0.02 *
Stretched exponential	DDC	0.1	0.02 *
	α	0.01 *	0.7
Kurtosis	D_K_	0.2	0.02 *
	K	0.03 *	0.07
Statistical	D_s_ (mode)	0.2	0.1
	σ (scale parameter)	0.7	0.2
	D_s'_ (mean)	0.2	0.02 *
	σ' (s.d.)	0.5	0.07
Bi-exponential	D	0.9	0.01 *
	*f*	0.0003 *	0.9
	D*	0.002 *	0.4
	*f*D*	0.3	0.05

**Fig. 4:**
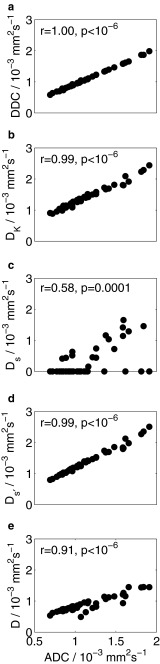
Correlation between ADC and **a** DDC from the stretched exponential model, **b** D_K_ from the kurtosis model, **c** D_s_ from the statistical model, **d** D_s'_ from the statistical model, and **e** D from the bi-exponential model

Assessment of correlation between ADC and other diffusion coefficients showed that DDC, D_K_, D_s'_ (mean), and D were strongly correlated with ADC (Fig. 4, r > 0.9, *p* < 10^-6^ in all cases). Correlation between D_s_ (mode) and ADC was less strong (r = 0.58, *p* = 0.0001). When assessing correlation of parameters within each model, no correlation was observed between α and DDC from the stretched exponential model, σ and D_s_ from the statistical model, nor between either *f* and D or D* and D from the bi-exponential model (Table 3). Weak correlation was observed between *f* and D* and between *f*D* and D from the bi-exponential model. Negative correlation was observed, however, between K and D_K_ from the kurtosis model, and strong positive correlation was observed between σ' and D_s'_ from the reformulated statistical model.

**Table 3:** Correlation between fitted parameters within each non-mono-exponential model

Model	Parameters	Pearson's correlation coefficient (r)	*p*-value
Stretched exponential	DDC, α	0.14	0.4
Kurtosis	D_K_, K	-0.72	<10^-6^ *
Statistical	D_s_ (mode), σ (scale parameter)	-0.13	0.4
	D_s'_ (mean), σ' (s.d.)	0.91	<10^-6^ *
Bi-exponential	D, *f*	0.28	0.08
	D, D*	0.18	0.2
	D*, *f*	-0.37	0.02 *
	D, *f*D*	0.64	5x10^-6^ *

Table shows Pearson’s linear correlation coefficient (r) and *p*-value for each pair of parameters. * *p* < 0.05

## Discussion

The preference for non-mono-exponential models in the majority of tumours indicates that these models provide a better description of the DW-MRI data in cervical tumours than a mono-exponential model. The absence of a clear preference for a particular model within each grade and type of tumour indicates that the models assessed here are applicable across all tumours and the choice of model is not driven purely by characteristics of a particular tumour grade or type. The preference for a non-mono-exponential model fit for the data from these tumours is consistent with results from other studies: a study in squamous cell carcinoma of the head and neck demonstrated that the kurtosis model provided a better fit to DW-MRI data than the mono-exponential model in primary tumours and in metastatic lymph nodes [7] and a study in advanced ovarian cancer demonstrated a preference for the stretched exponential model over the mono-exponential model in the majority of primary and metastatic lesions in DW-MRI data both pre- and post-treatment [11]. Clinical studies comparing various models for DW-MRI data from prostate cancer have also demonstrated a better fit in malignant as well as benign regions using the bi-exponential model [6] or the kurtosis model [17] compared with the mono-exponential model. In contrast, a study in normal and malignant breast tissue found that DW-MRI data from approximately half of all voxels preferred a mono-exponential model while either a stretched exponential or bi-exponential model was preferred in just under half of the voxels analysed [28]. It is, however, important to note that the mono-exponential model can be considered a special case of the other models applied here (using e.g. α = 1, K = 0, *f* = 0), as has been pointed out in previous studies [13], suggesting that tumours that prefer the mono-exponential model are not disadvantaged by the use of other models, although the additional parameters would be redundant if the mono-exponential model were preferred in all tumours. The stretched exponential, kurtosis, and statistical models describe the departure of the DW-MRI signal decay from mono-exponential behaviour, which may be due to intra-voxel heterogeneity or non-Gaussian diffusion processes, either of which may be related to the observed separation of types and grades of tumours.

The differences in fitted parameters between types or grades of tumours observed in this study indicate that these parameters are sensitive to micro-structural properties of the tumours. In this study, tumour grade was reflected by the diffusion coefficients; this was the case whether considering simply ADC or the diffusion coefficients from non-mono-exponential models (DDC, D_K_, D_s'_, and D). ADC could, therefore, be considered sufficient to describe tumour grade, but other parameters from the non-mono-exponential models (α, K, *f*, D*) provided further information that differentiated between tumour types. The use of non-mono-exponential models thus provides a more complete characterisation of the tumours than provided by ADC alone. The lower ADC observed in poorly differentiated tumours compared with well/moderately differentiated tumours is in agreement with results of previous studies [21, 22]. Diffusion coefficients from non-mono-exponential models (DDC, D_K_, D_s'_, and D) showed similar results to ADC, with lower estimates in poorly differentiated tumours compared with well/moderately differentiated tumours. These results, together with the strong correlation between ADC and these other diffusion coefficients, indicate that these parameters provide similar information to ADC. The parameter D_s_ from the statistical model was an exception to this behaviour, exhibiting only weak correlation with ADC and no significant difference between tumour grades. Median estimates of D_s_ included many values close to zero, as described in other studies [29]. After reformulating D_s_ to D_s'_, however, a significant difference between tumour grades was observed, and the median estimates matched ADC estimates more closely. It is notable that ADC and other diffusion coefficients differentiated grades of tumours where the major histological feature is increasing cell density and mitoses with increasing grade but that other parameters best differentiated squamous cell carcinoma and adenocarcinoma where structural features are more profound and rely on cell organisation and glandular architecture rather than cell density alone. Unfortunately, only two poorly differentiated adenocarcinomas were present in this cohort of patients due to the lower numbers of adenocarcinomas compared with squamous cell carcinomas in cancers of the uterine cervix [30].

Considering correlation of parameters within models, the absence of correlation between α and DDC from the stretched exponential model, σ and D_s_ from the statistical model, and either *f* and D or D* and D from the bi-exponential model indicates that these parameters provide complementary information and may be sensitive to different aspects of the tumour micro-structure. The strong correlation observed between σ' and D_s'_ from the reformulated statistical model, however, indicates that the additional parameter σ' may not provide additional information. The correlation between K and D_K_ from the kurtosis model indicates that these parameters also provide largely the same information, but in this case the additional parameter does provide some additional information as K and D_K_ were sensitive to different properties (type and grade, respectively).

Repeatability of fitted parameters is an important consideration in model selection. It was not possible to carry out repeated examinations on the patients in this study owing to the invasive nature of the MRI examination using an endovaginal receiver coil and it was, therefore, not possible to assess the test-retest repeatability of the parameter estimates. However, the repeatability of fitted parameters of the bi-exponential and stretched exponential models, which were preferred by largest numbers of tumours in this study, have been investigated in several previous studies that use similar DW-MRI protocols to the one used here [9, 11, 13, 31]. The parameters of the stretched exponential model have been shown to have very good repeatability (coefficient of variation (CV) ∼ 4 to 7 %), comparable to the repeatability of ADC estimates [9, 11, 13], while the repeatability of parameters from the bi-exponential model, particularly *f* and D* are significantly worse (CV > 20 %) [9, 11, 13, 31]. Whilst the ANOVA *p*-values indicate that *f* and D* show greater significance than α and K when making group comparisons between tumour type, the poorer repeatability of *f* and D* suggest that α and K may be more appropriate for differentiating tumour types in individuals.

An apparent limitation of this study is the use of a maximum b-value of 800 s mm^-2^ when applying models that were originally developed over much wider ranges of b-values [4, 5]. A maximum b-value of 800 s mm^-2^ is, however, typical of DW-MRI protocols in body applications where higher b-values increase the minimum TE attainable, thus lowering signal-to-noise ratio (SNR), and increase image distortion due to eddy current effects. The lack of b-values over 1000 s mm^-2^ would be a severe limitation in investigations of intra- and extra-cellular compartments in the brain, where some of the models investigated here were originally developed [4, 5]. In this study, however, the modelling process was a phenomenological investigation in order to assess the best description of the data and the original theoretical formulations were not implied. Also, although grading of tumours was carried out on cone biopsy in 30/42 cases, this represented a significant volume of tumour and mitigates against variations in tumour grade on needle biopsy.

In conclusion, non-mono-exponential models were preferred to the mono-exponential model in DW-MRI data from cervical tumours. ADC and the diffusion coefficients from other models were significantly different between grades of tumours, but showed no differences between tumour types, while α from the stretched exponential model, K from the kurtosis model, and *f* and D* from the bi-exponential model were significantly different between squamous cell carcinomas and adenocarcinomas. These results show that parameters from non-mono-exponential models are related to different aspects of tumour microstructure. Parameters from non-mono-exponential models may, therefore, have utility in probing features of tumour phenotype, which may be indicative of poor prognosis or progressive disease. Of the models tested, the stretched exponential model exhibited uncorrelated parameters, which were related to histological features and is, therefore, likely to be most relevant for clinical use.

## Abbreviations

DW-MRI: Diffusion-weighted magnetic resonance imaging
ADC: Apparent diffusion coefficient
EPI: Echo-planar imaging
FOV: Field-of-view
PE: Phase encoding
RL: Right-left
TE: Echo time
TR: Repetition time
SPIR: Spectral inversion recovery
NSA: Number of signal averages
ROI: Region of interest
DDC: Distributed diffusion coefficient
IVIM: Intravoxel incoherent motion
BIC: Bayesian information criterion
ANOVA: Analysis of variance
CV: Coefficient of variation
SNR: Signal-to-noise ratio
